# Comparison of the effects of introducing the CRISPR/Cas9 system by microinjection and electroporation into porcine embryos at different stages

**DOI:** 10.1186/s13104-020-05412-8

**Published:** 2021-01-06

**Authors:** Quynh Anh Le, Fuminori Tanihara, Manita Wittayarat, Zhao Namula, Yoko Sato, Qingyi Lin, Koki Takebayashi, Maki Hirata, Takeshige Otoi

**Affiliations:** 1 Laboratory of Animal Reproduction, Faculty of Bioscience and Bioindustry, Tokushima University, 2272-1 Ishii, Myozai-gun, Tokushima, 779-3233 Japan; 2 Faculty of Veterinary Science, Prince of Songkla University, Songkhla, Thailand; 3 College of Coastal Agricultural Sciences, Guangdong Ocean University, Guangdong, China; 4 School of Biological Science, Tokai University, Sapporo, Japan

**Keywords:** *B4GALNT2*, Electroporation, Microinjection, Pig, Xenotransplantation

## Abstract

**Objective:**

Cytoplasmic microinjection and electroporation of the CRISPR/Cas9 system into zygotes are used for generating genetically modified pigs. However, these methods create mosaic mutations in embryos. In this study, we evaluated whether the gene editing method and embryonic stage for gene editing affect the gene editing efficiency of porcine embryos.

**Results:**

First, we designed five guide RNAs (gRNAs) targeting the *B4GALNT2* gene and evaluated mutation efficiency by introducing each gRNA with Cas9 protein into zygotes by electroporation. Next, the optimized gRNA with Cas9 protein was introduced into 1-cell and 2-cell stage embryos by either microinjection or electroporation. The sequence of gRNA affected the bi-allelic mutation rate and mutation efficiency of blastocysts derived from electroporated embryos. Microinjection significantly decreased the cleavage rates in each embryonic stage and blastocyst formation rates in 2-cell stage embryos compared with electroporation (*p* < 0.05). However, the bi-allelic mutation rate and mutation efficiency of blastocysts from the 1-cell stage embryos edited using microinjection were significantly higher (*p* < 0.05) than those of blastocysts from the 2-cell stage embryos edited by both methods. These results indicate that the gene editing method and embryonic stage for gene editing may affect the genotype and mutation efficiency of the resulting embryos.

## Introduction

Genetically modified pigs are expected to be an excellent disease model contributing to human medicine [1, 2] and to be ideal organ donors for human xenotransplantation [3, 4]. The clustered regularly interspaced short palindromic repeats (CRISPR)/CRISPR-associated gene (CRISPR/Cas) system has been recently used to generate gene-modified animals carrying site-specific mutations by delivering the Cas9/gRNA complex into the embryos mostly via microinjection [5–7]. However, the genotype of the mutant embryos often exhibits a mosaic pattern, i.e. these mutant embryos are composed of several types of cells with different mutations [8–11]. In our previous study, we developed the GEEP (gene editing by electroporation of Cas9 protein) method that enabled disruption of the targeted genes with high-efficiency in pigs by introduction of CRISPR/Cas9 system into zygotes using electroporation [12]. GEEP does not cause the damage associated with micromanipulation, however, also induces the mosaic pattern in the embryos [13]. The occurrence of genetic mosaics including wild-type (WT) cells complicates the analysis of phenotype in the resulting pigs, therefore mosaicism is a serious problem.

It has been suggested that mosaic mutants arise due to Cas9/gRNA complexes that remain active throughout several cell divisions, or delayed mRNA expression, possibly triggered by cell division [11]. The continuous function of Cas9 on the targeting site during embryonic development induces the mosaic phenomenon of modification. On the other hand, the generation of mosaic mutants by introduction of Cas9 protein/gRNA complexes into 1-cell stage embryos depends on the time window between fertilization and the first DNA replication [14]. Moreover, Gu et al. [15] suggested that major zygotic genome activation with an open chromatin state occurs during the extended G2 phase of the 2-cell stage embryos, resulting in decreased mosaic phenomenon after gene editing by the CRISPR/Cas9 system.

β-1,4-N-acetyl-galactosaminyltransferase 2 (*B4GALNT2*) synthesizes carbohydrate xenoantigens, which account for the majority of human anti-pig antibody reactivity [16]. The generation of *B4GALNT2*-deficient animals appears to be a necessary step in achieving successful pig-human xenotransplantation. In the present study, to reduce the mosaic mutation in the early embryos, we evaluated the developmental competence and gene editing efficiency of porcine embryos at the 1-cell and 2-cell stage after introduction of the complexes of Cas9 protein and gRNA targeting the *B4GALNT2* gene by microinjection or electroporation methods.

## Main text

### Materials and methods

#### Oocyte collection, in vitro maturation(IVM) and in vitro fertilization (IVF)

Oocyte collection, IVM and IVF were performed as described previously [17]. Briefly, pig ovaries were obtained from prepubertal crossed gilts at a local slaughterhouse. Cumulus-oocyte complexes (COCs) were collected and cultured in maturation medium. After IVF, the putative zygotes were cultured in porcine zygote medium (PZM-5; Research Institute for the Functional Peptides Co., Yamagata, Japan) until microinjection and electroporation treatments.

#### Electroporation

Electroporation was performed as described previously [12]. Briefly, embryos were electroporated (five 1-ms pulses at 25 V) with Nuclease-Free Duplex Buffer (Integrated DNA Technologies, Coralville, IA, USA) containing 100 ng/μl of gRNA (Alt-R™ CRISPR crRNAs and tracrRNA) (Integrated DNA Technologies) and 100 ng/µl Cas9 protein (Takara Bio, Inc., Shiga, Japan). After electroporation, the embryos were cultured in PZM-5. At Day 3 after fertilization (Day 0), all of the cleaved embryos were subsequently cultured in porcine blastocyst medium (PBM; Research Institute for the Functional Peptides Co.) for 4 days.

#### Cytoplasmic microinjection

The CRISPR/Cas9 components were injected into 1-cell and 2-cell stage embryos in a 20 μl drop of PZM-5 covered by mineral oil. The duplex buffer containing 100 ng/μl of gRNA and 100 ng/μl of Cas9 protein was loaded into the injection pipette (Femtotips II, Eppendorf, Hamburg, Germany) and injected into the cytoplasm by air pressure using a microinjector (FemtoJet 4i; Eppendorf). After microinjection, the embryos were cultured in PZM-5 and PBM as described above.

#### Analysis of targeted gene sequence after microinjection and electroporation

Genomic DNA isolated from resulting blastocysts collected individually were subjected to polymerase chain reaction (PCR) using the specific primers (Additional file 1: Table S1). The PCR products were analyzed by Sanger sequencing and the TIDE (tracking of indels by decomposition) bioinformatics package [18] as described previously [19]. Blastocysts were classified as having bi-allelic mutations (carrying no WT sequences), mosaics (carrying more than one type of mutation and the WT sequence), or WT (carrying only the WT sequence).

### Experimental design

#### Experiment 1: Comparison of gRNA gene-targeting efficiency

To confirm the optimal gRNA for efficient gene editing, we designed five gRNAs (#1–#5) targeting different sites of the *B4GALNT2* gene (Additional file 2: Table S2). Each gRNA and Cas9 protein were introduced into porcine embryos by electroporation at 12 h after the start of IVF. The blastocyst formation rate from the embryos post introduction of each gRNA and the mutation efficiency in the resulting blastocysts were evaluated, as described above. As a control, some embryos were cultured with PZM-5 and PBM for 7 days without electroporation treatment.

#### Experiment 2: Comparison of the development stage and gene editing method

Embryos at the 1-cell and 2- cell stages were collected at 12 h and 24 h after the start of IVF, respectively. gRNA #1, which was confirmed by Experiment 1 to show high-efficiency targeting of *B4GALNT2* gene, was used in Experiment 2. Cas9 protein with gRNA #1 was introduced into the embryos at each stage by microinjection and electroporation. For the 2-cell stage embryos, we injected Cas9 protein with gRNA into both blastomeres, separately. As a control, the embryos at the 1-cell and 2-cell stages were microinjected and electroporated without gRNA and Cas9. After the in vitro culture, the resulting blastocysts were collected and subjected to analysis of genotype, as described above.

#### Statistical analysis

All percentage data were subjected to arcsine transformation before performing analysis of variance (ANOVA). The transformed data were tested by ANOVA, followed by Fisher’s protected least significant difference test, using StatView software (Abacus Concepts, Berkeley, CA, USA). The percentages of mosaic and bi-allelic blastocysts in the total number of blastocysts were analyzed by chi-squared analysis with Fisher's exact test. Differences with a probability value (*p*) of 0.05 or less were considered statistically significant.

### Results

#### Experiment 1

**Fig. 1:**
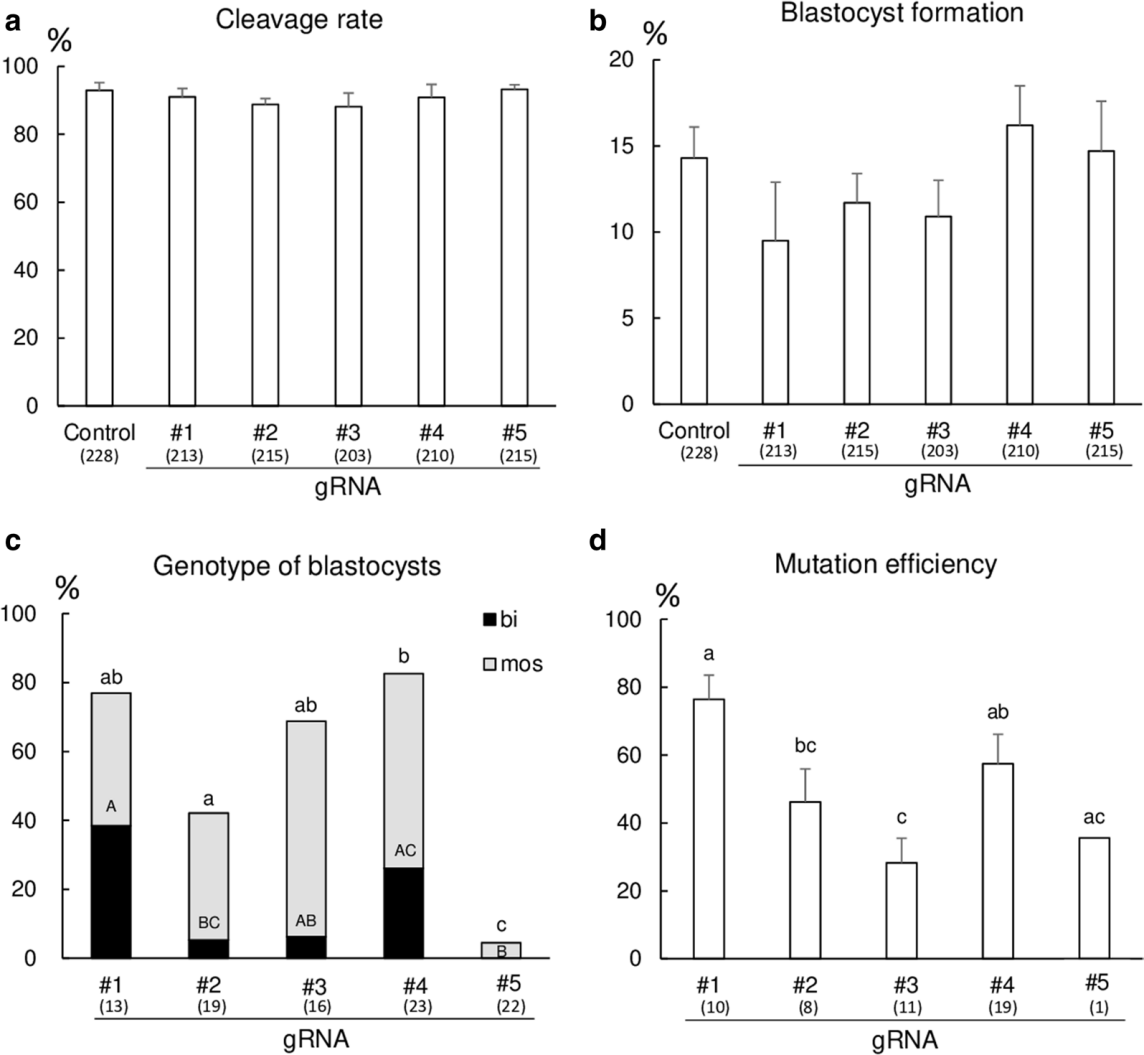
Introduction of the CRISPR/Cas9 system targeting the *B4GALNT* gene into embryos by electroporation. **a** Cleavage and **b** blastocyst formation rates of electroporated embryos. Five replicates were analyzed for each treatment group. **c** Genotype of blastocysts, determined using Sanger sequencing and TIDE analysis. The proportions were calculated by dividing the number of gene-edited (bi-allelic and mosaic) blastocysts by the total number of sequenced blastocysts. bi: blastocysts having bi-allelic mutations, mos: blastocysts having mosaic mutations.* d* Mutation efficiency of gene-edited blastocysts. The mean proportions represent the proportion of indel mutation events in gene-edited blastocysts. The numbers within parentheses under the X-axis indicate the total number of examined samples. Error bars indicate mean ± SEM (**a**, **c**). ^a−c, A−C^Bars with different letters differ significantly (*P* < 0.05)

Representative images of the genotyping are shown in Additional file 3: Figure S1. There were no significant differences in the cleavage and blastocyst formation rates of embryos edited by electroporation among the different gRNA groups (Fig. 1a, b). The total mutation rate of blastocysts derived from the embryos electroporated with gRNA #4 significantly increased (*p* < 0.05) compared with that with gRNAs #2 and #5 (Fig. 1c). The bi-allelic mutant rate of blastocysts from embryos electroporated with gRNA #1 was significantly higher (*p* < 0.05) than that with gRNAs #2 and #5. Moreover, the mutation efficiency in gene-edited blastocysts derived from embryos electroporated with gRNA #1 significantly increased (*p* < 0.05) compared with that with gRNAs #2 and #3 (Fig. 1d).

#### Experiment 2

As shown in Table 1, the cleavage rates of embryos treated using the microinjection method significantly decreased compared with those of embryos treated using the electroporation method, irrespective of the embryonic stage (*p* < 0.05). The blastocyst formation rate of 1-cell stage and 2-cell stage embryos having Cas9 and gRNA introduced via microinjection was significantly lower (*p* < 0.05) than that of 2-cell stage embryos having gRNA and Cas9 introduced via electroporation. When the embryonic stage and gene editing method were same, the cleavage rates and blastocyst formation rates of embryos treated with gRNA and Cas9 were statistically same as that of embryos treated without gRNA and Cas9.

**Table 1:** Introduction of CRISPR/Cas9 system into embryos at different stages by cytoplasmic microinjection and electroporation

Embryonic stage	Gene editing method	Cas9/gRNAcomplex*	No. of embryos examined	No. (%) of embryos	
				Cleavage**	Developed to blastocysts
1-cell	MI	−	201	138 (68.7 ± 2.6)^a^	21 (10.5 ± 1.7)^a^
		+	248	155 (62.4 ± 2.9)^a^	20 ( 8.1 ± 1.1)^a^
	EP	−	210	192 (91.5 ± 1.7)^b^	38 (18.1 ± 1.4)^ab^
		+	250	230 (92.0 ± 0.9)^b^	40 (16.0 ± 3.1)^ab^
2-cells	MI	−	105	78 (74.3 ± 1.7)^a^	19 (18.2 ± 1.8)^ab^
		+	119	73 (61.5 ± 6.2)^a^	15 (13.4 ± 6.4)^a^
	EP	−	105	91 (86.9 ± 1.9)^b^	34 (32.6 ± 1.9)^c^
		+	128	114 (89.7 ± 4.3)^b^	33 (26.3 ± 7.7)^bc^

*MI* microinjection, *EP* electroporation

All of the experiments were repeated four to five times. Data are expressed as the mean ± SEM

* The embryos at the 1-cell and 2-cell stages were microinjected and electroporated with (+) or without (−) gRNA and Cas9 complexes

** The embryos treated at 2-cell stage were defined as cleavage when they have more than 3 blastomeres on day 7

^a−c^Values with different superscripts in the same column are significantly different (*p* < 0.05)

**Fig. 2:**
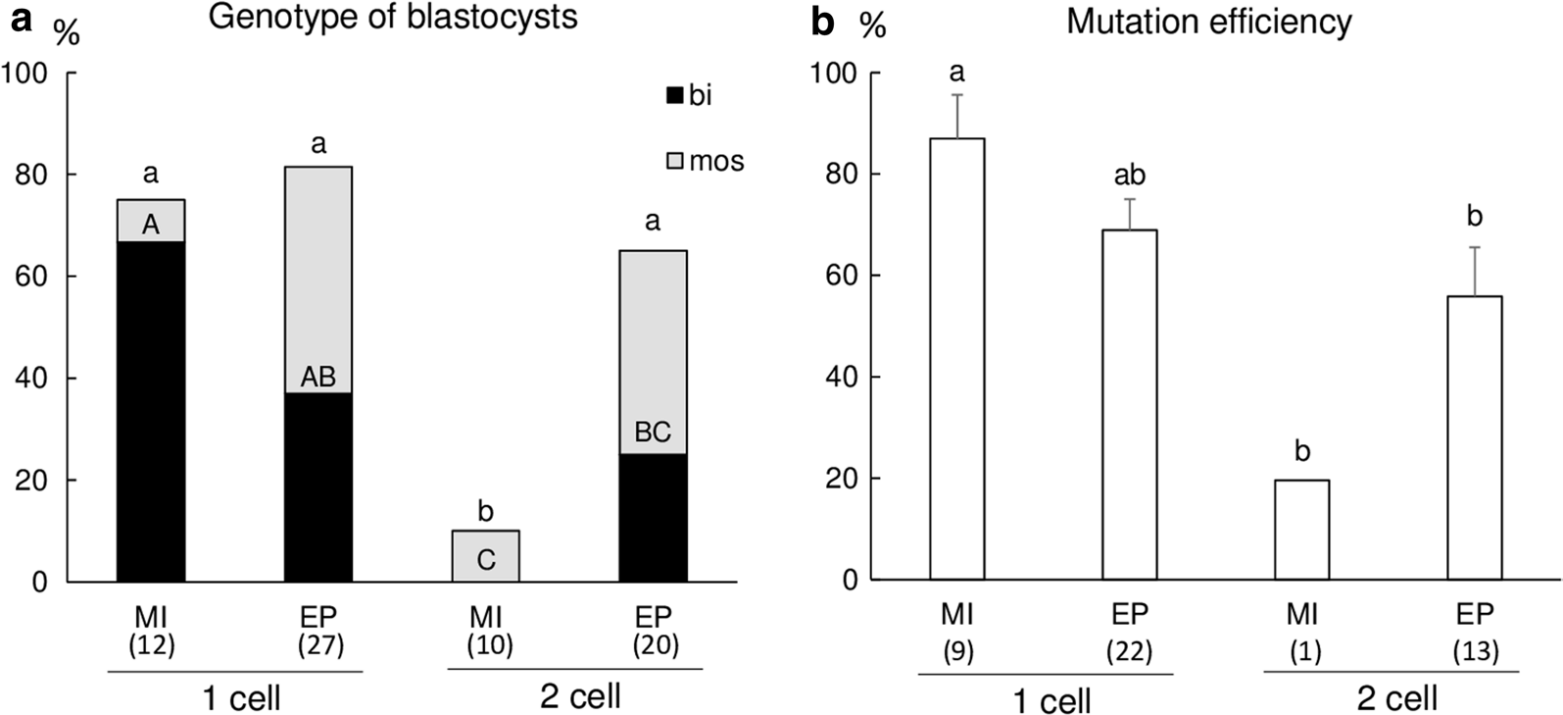
Introduction of the CRISPR/Cas9 system into embryos at different development stages by microinjection and electroporation. **a** Genotype of blastocysts. The proportions were calculated by dividing the number of gene-edited (bi-allelic and mosaic) blastocysts by the total number of analyzed blastocysts. bi: blastocysts having bi-allelic mutations, mos: blastocysts having mosaic mutations. **b** Mutation efficiency of gene-edited blastocysts. The mean proportions represent the proportion of indel mutation events in gene-edited blastocysts. The numbers within parentheses under the X-axis indicate the total number of examined samples. Error bars indicate mean ± SEM **b**. ^a−b, A−C^Bars with different letters differ significantly (*P* < 0.05). MI: microinjection, EP: electroporation

The total mutation rate of blastocysts derived from the 2-cell stage embryos edited using the microinjection method significantly decreased (*p* < 0.05) compared with that of the other treatment groups (Fig. 2a). The rates of bi-allelic mutant and mutation efficiency of blastocysts from the 1-cell stage embryos edited using the microinjection method were significantly higher (*p* < 0.05) than those of blastocysts from the 2-cell stage embryos edited using both methods (Fig. 2a, b).

### Discussion

Xenotransplantation is a potential solution to address the growing demand for organs suitable for transplantation. *B4GALNT2* synthesizes carbohydrate xenoantigen, which is one of the major xenoantigen expressed at high levels in porcine tissue but absent in human tissue [16]. Therefore, generating *B4GALNT2*-deficient pigs is the first step for achieving successful pig-to-human xenotransplantation. To date, highly efficient gene modification of embryos using the CRISPR/Cas9 system introduced by microinjection and electroporation has been reported in experimental animals [6, 12, 20]. However, mosaicism including WT cells is a serious problem for gene modification by the CRISPR/Cas9 system [21]. One-step generation of F0 pigs with the completely desired gene modification saves cost and time.

We first optimized the gRNA for efficient targeting of the *B4GALNT2* gene using the electroporation method. Although we could not evaluate the quality of blastocysts because we subjected all blastocysts derived from electroporated embryos for genotyping, we found that the sequence of gRNA used in this study did not affect the blastocyst formation rate after the electroporation treatment. However, the mutation efficiency of resulting blastocysts was affected by the sequence of gRNA. These results were supported by previous studies that demonstrated the design of gRNA to be one of the keys factors enabling gene-targeting and mutation efficiency in the CRISPR/Cas9 system [22, 23].

In Experiment 2, we compared the effects of the gene editing method and embryonic stage on the development and mutation efficiency of porcine embryos. Our results demonstrated that the gene editing method affected the cleavage rates. The blastocyst formation rate of the 2-cell stage embryos electroporated in experiment 2 was approximately two times higher than that of the control embryos (experiment 1). The higher blastocyst formation rate seems to be due to the use of only embryos reaching the 2-cell stage for electroporation. However, the mean rates of blastocyst formation in the microinjection method decreased to approximately half of that in the electroporation method, irrespective of the embryonic stage. One possible explanation for the decrease in blastocyst formation rates is that mechanical invasion during the microinjection procedure of the CRISPR/Cas9 system may reduce the developmental competence of embryos [24, 25]. Another cause of reduced embryo development may be the amount of expressed protein and toxicity that depends on the Cas9 concentration injected. However, investigation of embryonic development has revealed that injection of 200 ng/µl Cas9 mRNA is nontoxic to embryos [26]. In the present study, we injected only 100 ng/µl Cas9 protein (160 kDa), which is much smaller than Cas9 mRNA (~ 1500 kDa), suggesting low toxicity. In Experiment 2, the blastocyst formation rate was affected by microinjection treatment, but we could not evaluate the quality of blastocysts because of genotyping analysis. A previous study using parthenogenetic embryos demonstrated that cytoplasmic microinjection reduced blastocyst formation rate, but the average cell number of blastocysts in the microinjected group was comparable to that in the untreated control group [27]. We guess that the quality of blastocysts derived from microinjected embryos may be also comparable to that in the control and electroporated group in this study.

Our results demonstrated that the embryonic stage effect on the mutation of blastocysts was apparent in the microinjection method, in which blastocysts from the 1-cell stage embryos had higher rates of total mutation, bi-allelic mutation, and mutation efficiency compared with blastocysts from the 2-cell stage embryos. However, these results were not consistent with the findings of Gu et al. [15], who reported that, in mice, the knock-in efficiency by 2-cell microinjection of CRISPR reagents was higher than that by zygote microinjection. They suggested that major zygotic genome activation, which takes place during the extended G2 phase of the 2-cell stage, is associated with an open chromatin state, resulting in increase of the accessibility of the chromatin to editing enzymes and repair templates. In contrast, introduction of the CRISPR/Cas9 system by electroporation into early 1-cell stage embryos prior to or soon after the first cleavage divisions has been shown to generate high non-mosaic mutants in mouse embryos [14]. However, the extent of mosaicism varies from embryo to embryo and from gene to gene [15]. Therefore, the discrepancy in the embryonic stage effects remains to be explained, but it might be partly due to the differences in animal species or target genes.

The mosaic issue remains to be resolved for genetically engineering large animal models. Therefore, to obtain highly efficient gene-edited embryos for one-step generation, microinjection of the CRISPR/Cas9 system into 1-cell stage embryos may be suitable in pigs. However, The minimization of the embryonic damage and conservation of gene expression with high levels following transfection affects the success of gene editing by CRISPR/Cas9 system. Therefore, gene editing via the electroporation method may have a benefit as an alternative method when the viability of the embryos is a priority.

## Limitations

One limitation in this study was that only *B4GALNT2* gene was targeted due to the complicated experimental procedures. Different target genes should be compared in future studies.

## Supplementary Information

**Additional file 1: Table S1.** Primer sequenences used for sequencing analysis.

**Additional file 2: Table S2.** Sequences of gRNA targeting *B4GALNT* gene.

**Additional file 3: Figure S1.** Representative images of genotyping. (a) Representative image of electrophoresis. (b) Representative genomic sequences of porcine blastocysts derived from zygotes electroporated with Cas9 and different gRNAs targeting the *B4GALNT* gene. WT: Wild-type control, M: 100-bp DNA Ladder.

## Data Availability

The datasets used and/or analyzed during the current study are available from the corresponding author on reasonable request.

## Abbreviations

ANOVA: Analysis of variance
B4GALNT2: β-1,4-N-Acetyl-galactosaminyltransferase 2
CRISPR: The clustered regularly interspaced short palindromic repeats
Cas: CRISPR-associated gene system
COCs: Cumulus-oocyte complexes
GEEP: Gene editing by electroporation of Cas9 protein
IVF: In vitro fertilization
IVM: In vitro maturation
PBM: Porcine blastocyst medium
PCR: Polymerase chain reaction
PZM-5: Porcine zygote medium
TIDE: Tracking of indels by decomposition
WT: Wild-type
