# Supplementary material for: Comparison of the effects of introducing the CRISPR/Cas9 system by microinjection and electroporation into porcine embryos at different stages
Source: BMC Res Notes. 2021 Jan 6;14:7. doi: 10.1186/s13104-020-05412-8 (PMC7788904; doi:10.1186/s13104-020-05412-8)
Supplement: Supplementary file 1 — Additional file 1: Table S1. Primer sequenences used for sequencing analysis. [file 13104_2020_5412_MOESM1_ESM.docx]

Additional fle 1: Table S1. Primer sequenences used for sequencing analysis

| **gRNA** | **Forward primer** | **Reverse primer** |
| --- | --- | --- |
| #1 | 5'-GACCAGACATCGTTCCCAGT-3' | 5'-GGGAACTGGCTGTAAAGTGG-3' |
| #2 | 5'-GACCAGACATCGTTCCCAGT-3' | 5'-GGGAACTGGCTGTAAAGTGG-3' |
| #3 | 5'-TAGGGGGAAAAACACACTGG-3' | 5'-CACCCTCGGGAATGAGTAGA-3' |
| #4 | 5'-TAGGGGGAAAAACACACTGG-3' | 5'-CACCCTCGGGAATGAGTAGA-3' |
| #5 | 5'-TAGGGGGAAAAACACACTGG-3' | 5'-CACCCTCGGGAATGAGTAGA-3' |
