# Supplementary material for: Comparison of the effects of introducing the CRISPR/Cas9 system by microinjection and electroporation into porcine embryos at different stages
Source: BMC Res Notes. 2021 Jan 6;14:7. doi: 10.1186/s13104-020-05412-8 (PMC7788904; doi:10.1186/s13104-020-05412-8)
Supplement: Supplementary file 2 — Additional file 2: Table S2. Sequences of gRNA targeting B4GALNT gene. [file 13104_2020_5412_MOESM2_ESM.docx]

Additional file 2: Table S2. Sequences of gRNA targeting *B4GALNT* gene

| **gRNA** | **Sequence** | **PAM** | **Strand** | **Position** |
| --- | --- | --- | --- | --- |
| #1 | ttgaggatcgacagacatct | AGG | Antisense | Exon 2 |
| #2 | acataaagagtccaacgctc | AGG | Antisense | Exon 2 |
| #3 | gatgcccgaaggcgtcacat | TGG | Antisense | Exon 3 |
| #4 | gtctcctcaggttcactgcg | GGG | Antisense | Exon 3 |
| #5 | atgtgacgccttcgggcatc | AGG | Sense | Exon 3 |
