# Supplementary material for: Comparison of the effects of introducing the CRISPR/Cas9 system by microinjection and electroporation into porcine embryos at different stages
Source: BMC Res Notes. 2021 Jan 6;14:7. doi: 10.1186/s13104-020-05412-8 (PMC7788904; doi:10.1186/s13104-020-05412-8)
Supplement: Supplementary file 3 — Additional file 3: Figure S1. Representative images of genotyping. (a) Representative image of electrophoresis. (b) Representative genomic sequences of porcine blastocysts derived from zygotes electroporated with Cas9 and different gRNAs targeting the B4GALNT gene. WT: Wild-type control, M: 100-bp DNA Ladder. [file 13104_2020_5412_MOESM3_ESM.docx]

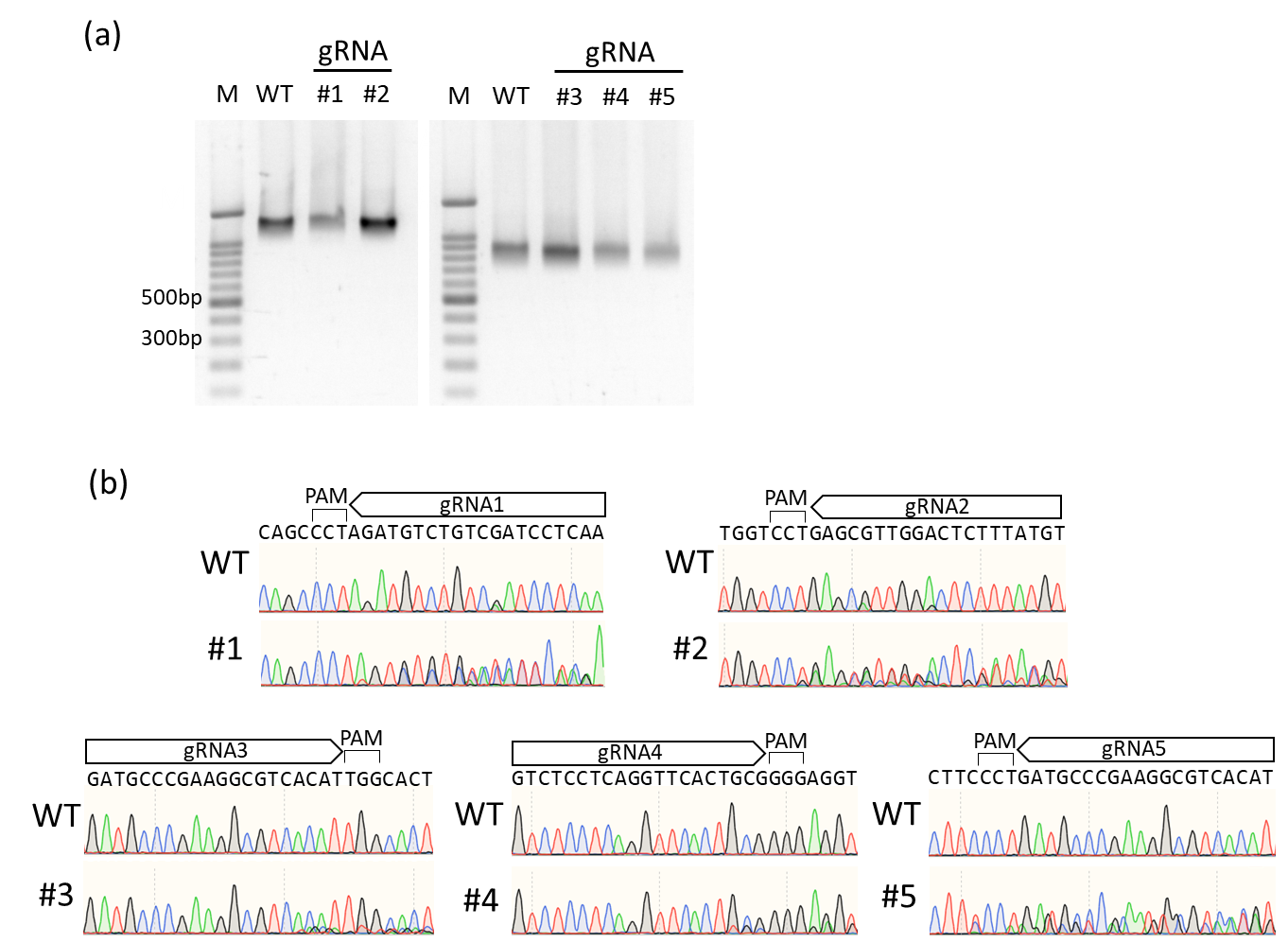


Additional file 3: Figure S1. Representative images of genotyping. (a) Representative image of electrophoresis. (b) Representative genomic sequences of porcine blastocysts derived from zygotes electroporated with Cas9 and different gRNAs targeting the *B4GALNT* gene. WT: Wild-type control, M: 100-bp DNA Ladder.
